# Liquid biopsy at the frontier of detection, prognosis and progression monitoring in colorectal cancer

**DOI:** 10.1186/s12943-022-01556-2

**Published:** 2022-03-25

**Authors:** Hui Zhou, Liyong Zhu, Jun Song, Guohui Wang, Pengzhou Li, Weizheng Li, Ping Luo, Xulong Sun, Jin Wu, Yunze Liu, Shaihong Zhu, Yi Zhang

**Affiliations:** 1 Department of General Surgery, Third Xiangya Hospital, Central South University, Changsha, 410013 China; 2 Department of General Surgery, Affiliated Hospital of Xuzhou Medical University, Xuzhou, 221000 China

**Keywords:** Circulating tumor cells, Circulating tumor DNA, Exosomes, Tumor-educated platelets, Clinical application, Colorectal cancer, Liquid biopsy

## Abstract

Colorectal cancer (CRC) is one of the most common cancers worldwide and a leading cause of carcinogenic death. To date, surgical resection is regarded as the gold standard by the operator for clinical decisions. Because conventional tissue biopsy is invasive and only a small sample can sometimes be obtained, it is unable to represent the heterogeneity of tumor or dynamically monitor tumor progression. Therefore, there is an urgent need to find a new minimally invasive or noninvasive diagnostic strategy to detect CRC at an early stage and monitor CRC recurrence. Over the past years, a new diagnostic concept called “liquid biopsy” has gained much attention. Liquid biopsy is noninvasive, allowing repeated analysis and real-time monitoring of tumor recurrence, metastasis or therapeutic responses. With the advanced development of new molecular techniques in CRC, circulating tumor cells (CTCs), circulating tumor DNA (ctDNA), exosomes, and tumor-educated platelet (TEP) detection have achieved interesting and inspiring results as the most prominent liquid biopsy markers. In this review, we focused on some clinical applications of CTCs, ctDNA, exosomes and TEPs and discuss promising future applications to solve unmet clinical needs in CRC patients.

**Supplementary Information:**

The online version contains supplementary material available at 10.1186/s12943-022-01556-2.

## Background

Colorectal cancer (CRC) is the second mainspring of cancer and cancer-related mortality globally [1]. The incidence and fatality rate are increasing consecutively year by year, seriously endangering people’s health. Current therapeutic approaches for CRC include endoscopic and surgical resection, systemic adjuvant chemotherapy, radiation therapy, targeted therapy and immunotherapy [1, 2]. Chemoresistance and tumor heterogeneity are the main reasons for tumor recurrence. Due to the poor response of many patients to current treatment strategies and because CRC survival is highly dependent upon early diagnosis and early treatment, a reliable biomarker that can predict the therapeutic response as early as possible is urgently needed. To date, tissue biopsy remains the gold standard for tumor identification. However, a major problem is that it is difficult to monitor disease progression through repeated biopsies due to repeated injury and poor patient compliance. Moreover, a single biopsy is usually not representative of a patient’s heterogeneity and cannot reflect the ever-changing complete cancer gene expression profile, and it is limited by the site of tissue removal, poor sensitivity and accuracy, and high procedural costs. Indeed, there is a critical need to find a minimally invasive method to screen the high-risk population and detect CRC presence in asymptomatic patients at an earlier stage and curable stage.

**Fig. 1:**
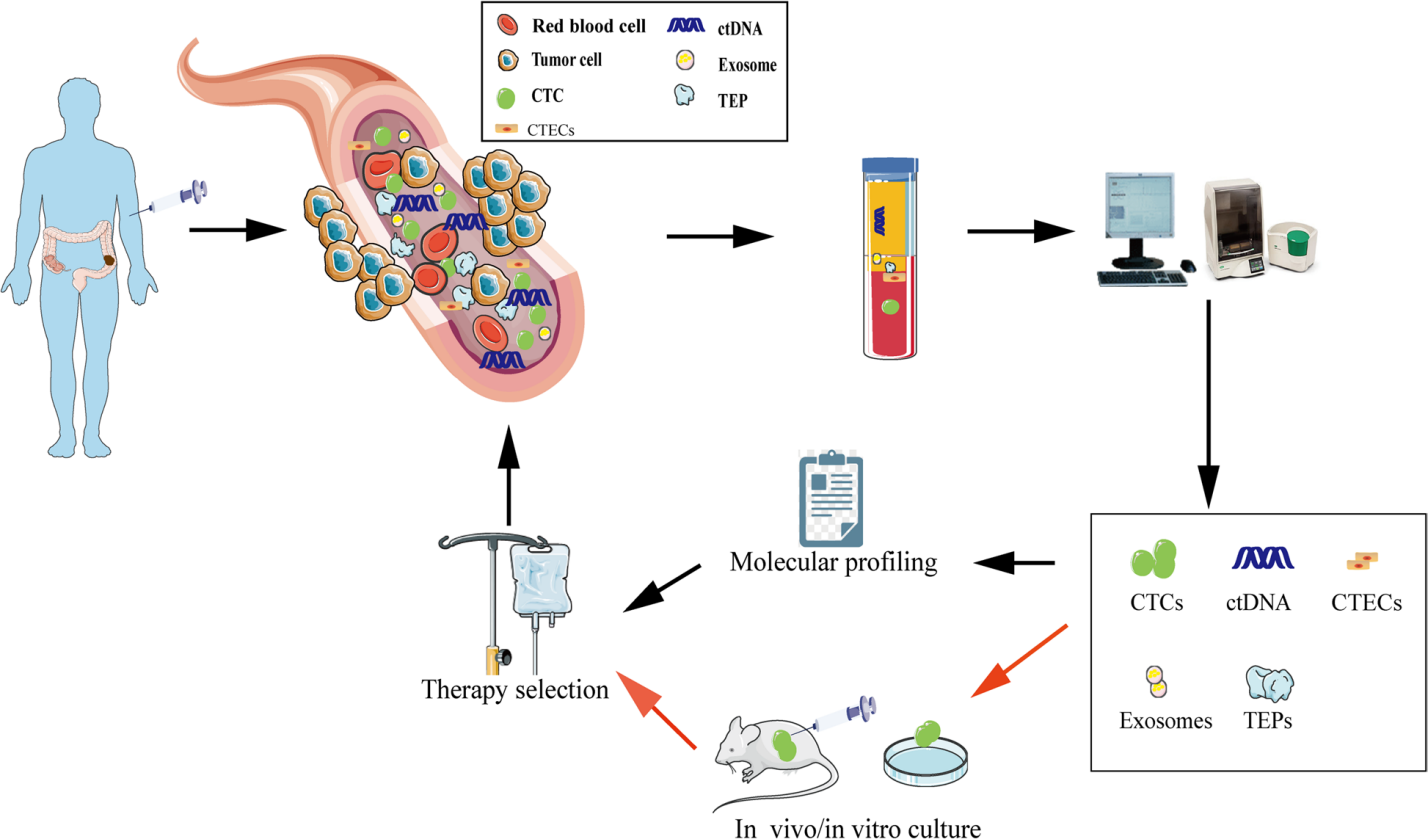
Liquid biopsy in CRC patients. CTCs, ctDNA, exosomes, CTECs, TEPs can all be detected by blood samples collected for liquid biopsy. Their analyses can be used to help with molecular profiling and treatment selection. CTCs can also be employed for culture and xenografting to help in CRC treatment selection

Owing to its invasive nature, routine biopsy cannot always be done routinely. Even though a single biopsy can catch a limited snapshot of the tumor, it always fails to reflect its temporal heterogeneity. This dilemma has opened a new diagnostic avenue: liquid biopsy. A liquid biopsy collects samples of biological fluids such as blood, saliva, pleural fluid, ascites, stool, urine, and cerebrospinal fluid (CSF) for different analyses [3]. Liquid biopsy is a new technology to detect tumor-related molecular markers in specimens by analyzing CTCs, ctDNA, circulating free (cf) DNA or RNA, exosomes, TEPs, circulating tumor-derived endothelial cells (CTECs) and protein molecules. The possible sources of liquid biopsy are illustrated in Fig. 1. With the rapid development of cell separation technology and gene detection technology, the core position of liquid biopsy in tumor precision medicine has been increasingly highlighted. Compared with tissue biopsy, liquid biopsy technology can better overcome tumor heterogeneity and facilitate repeated detection. It can also conduct comprehensive real-time monitoring at the molecular level to understand the tumor load and genetic changes of patients in the whole process of the disease, which is also conducive to the selection and adjustment of follow-up treatment plans and has broad clinical application prospects. Although proteomics, exosome, and miRNA research are still in its infant stage, their clinical application is limited. However, ctDNA and CTCs in peripheral blood have certain clinical value in the early prognosis of patients, monitoring recurrence, evaluating efficacy, and guiding precise treatment. Ongoing clinical trials are evaluating the use of liquid biopsy to guide therapeutic strategies in CRC patients and will ultimately shed light on whether liquid biopsy can be an effective tool for predicting the prognosis of CRC patients (Table S1).

In this review, how liquid biopsy opens a new avenue for CRC in detection, prognosis and progression monitoring was the focus. In addition to the epigenetic mechanisms involved in CRC, the concept and clinical applications of liquid biopsy were described. Additionally, a review of the methodologies used to detect these epigenetic changes in liquid biopsy was provided, as well as a description of the clinical utility of epigenetic markers in liquid biopsy for the diagnosis of CRC patients. The current limitations and speculated future directions of liquid biopsy in CRC biology was also discussed.

## Main text

### History of liquid biopsy

The term “liquid biopsy” was originally introduced by Pantel and Alix-Panabieres to define the markers in the blood to reflect specific information about the tumor more than 10 years ago [4]. As a means to identify tumor-related changes in body fluids, CTCs, ctDNA, exosomes, and TEPs have been studied over the past few decades.

#### Circulating tumor cells

CTCs are rare cancer cells that have been shed into the vasculature from a primary or metastatic tumor and circulate in the peripheral blood [5]. Thomas Ashworth first discovered CTCs in the presence of plasma in a metastatic cancer patient in 1869, which paved the way for liquid biopsy [6]. CTCs, however, have been known for quite a while but little research has been conducted on them. Research conducted by Massimo Cristofanilli et al. in 2004 confirmed that CTCs are an independent prognostic factor in metastatic breast cancer [7]. Surprisingly, CTCs were found in early tumors until 2010. This suggests that CTC counts can be used in the early diagnosis of tumors [8]. Then, 2 years later, the CTC classification was used to guide precise treatment of breast cancer [9]. Generally, the low number of CTCs in the blood limits its development. However, CTC-whole exome sequencing (WES) and whole genome sequencing (WGS) overturn this limitation by highlighting the heterogeneity of cancer [10, 11].

#### Circulating free DNA

While cfDNA is mostly derived from normal healthy leukocytes and stromal cells, in cancer patients, cfDNA can also incorporate tumor-derived DNA, referred to as ctDNA. Floating DNA fragments from plasma were first detected as early as 1948 by Mandel and Metais [12]. Unfortunately, for a long time, this groundbreaking effort received little attention. In 1977, Leon et al. observed that the plasma free DNA level was significantly higher in tumor patients, particularly in advanced tumor patients, than in healthy individuals. As a result, it is speculated that free DNA may be strongly tied to malignancies [13]. However, it was not until 1989 that Stroun reported that some cfDNA in tumor patients was derived from tumor cells. As a result, research on cfDNA has progressed slowly in the past due to the lack of sensitive and specific experimental methods. In 1994, KRAS and NRAS mutation genes were identified in blood cfDNA from patients with pancreatic carcinoma and myelodysplastic syndrome or acute myelogenous leukemia, which may provide the basis for the diagnosis of tumors [14]. Then, methylation-specific PCR was used by M Esteller to search for promoter hypermethylation of tumor-related genes in 22 patients suffering from non-small-cell lung cancer in 1999. All tumor stages exhibited abnormal promoter methylation in serum DNA, suggesting that it may be useful to detect a recurrence of cancer or to monitor it for recurrence [15]. Despite extensive research, such studies have yet to be validated in clinical practice. Until 2005, the assessment of ctDNA mutations was offered for the first time to the clinic. Following 3 years, Diehl F et al. tracked the ctDNA of 18 patients with colorectal cancer and detected some hotspot mutation genes, such as APC, KRAS, TP53, and PIK3CA, and found that the ctDNA mutation rate changed with the treatment process [16]. The European Union’s EMA first approved the clinical validation of ctDNA to detect EGFR mutations in carcinoma in 2014, signalling the first time that ctDNA has been used in clinical practice. Liquid biopsy was endorsed in the National Comprehensive Cancer Network (NCCN) guidelines in 2016. With the progress of detection technology, ctDNA measurements will be extensively implemented in new therapies to appropriately monitor tumor burden dynamics and investigate acquired resistance to cancer treatments.

#### Exosomes

In general, extracellular vesicles (EVs) can be broadly classified into three categories: exosomes, micro-vesicles and apoptotic bodies [17]. Exosomes are EVs with a diameter of approximately 30–150 nm. They can be secreted into the body fluid by all living cells, including tumor and normal cells. Exosomes were first discovered by EG Trams in sheep reticulocytes in 1981 and named by Johnstone 6 years later [18]. G. Raposo discovered a kind of B cell-derived exosome that can directly participate in the antitumor response of CD4^+^ cells in 1996 [19]. T Two years later, L. Zitvogel et al. confirmed that DCs can also release exosomes, augmenting the T cell-dependent antitumor effect [20]. Since then, the study of exosomes has opened a new era. With the advancement of exosome research, it was recognized that they play an important role in antigen presentation, cell differentiation, growth, tumor immune response, tumor cell migration and invasion [21]. However, despite their many benefits, exosomes have certain limitations in clinical use, such as limited targeting efficiency and being quickly delivered by the immune system. Therefore, more research on exosomes should be conducted to remove the stumbling block of clinical applications.

#### TEPs

**Fig. 2:**
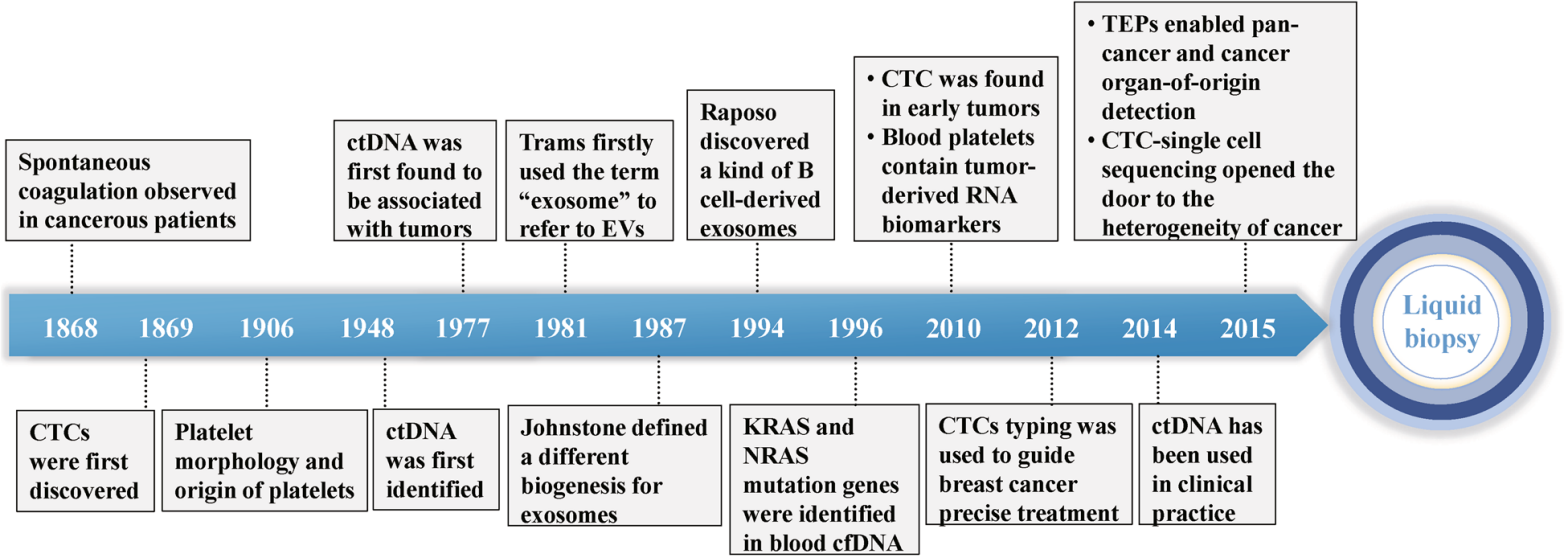
Timeline of key discoveries of liquid biopsy

Blood platelets, the second most prevalent cell type in peripheral blood, originate from megakaryocytes (MKs), which are recognized for their function in coagulation of blood and wound healing [22, 23]. Platelets can be regarded as “scanning soldiers” when immunological and inflammatory activity are associated with disease, such as tumor progression [24]. The relationship involving platelets and tumor growth and cancer progression has also been gradually discovered. Since Trousseau first shared the phenomenon that spontaneous coagulation is common in cancerous patients in 1865, activation of the clotting cascade via host-tumor interactions has been discovered in practically all different types of cancer [25]. Then, in 1906, Wright identified platelets separated from circulating megakaryocytes in cancer patients [26]. According to Nilsson et al. in 2010, platelets from cancer patients can pick up secreted RNA-containing membrane vesicles originating from human cancer cells [27]. Best et al. investigated the potential for TEP-based pan-cancer, multiclass cancer, and companion diagnostics by evaluating the platelet mRNA profiles of various cancer patients and healthy donors in 2015 [28]. Additionally, platelets have also been recognized as an effective participant in systemic and local responses to tumor growth and metastasis. TEPs are being used to bring platelets into contact with malignant cells. Considered together, CTCs, ctDNA, exosomes, and TEPs are prospective biomarkers for cancer diagnosis, screening, and therapy monitoring, and their practical translation would be contingent on the development of adequate isolation technologies. To summarize the evolution of liquid biopsy, it has been slow in the past (Fig. 2). However, with the rapid advancement of technology, liquid biopsy is poised to enter a new era.

### Circulating tumor cells in CRC

**Fig. 3:**
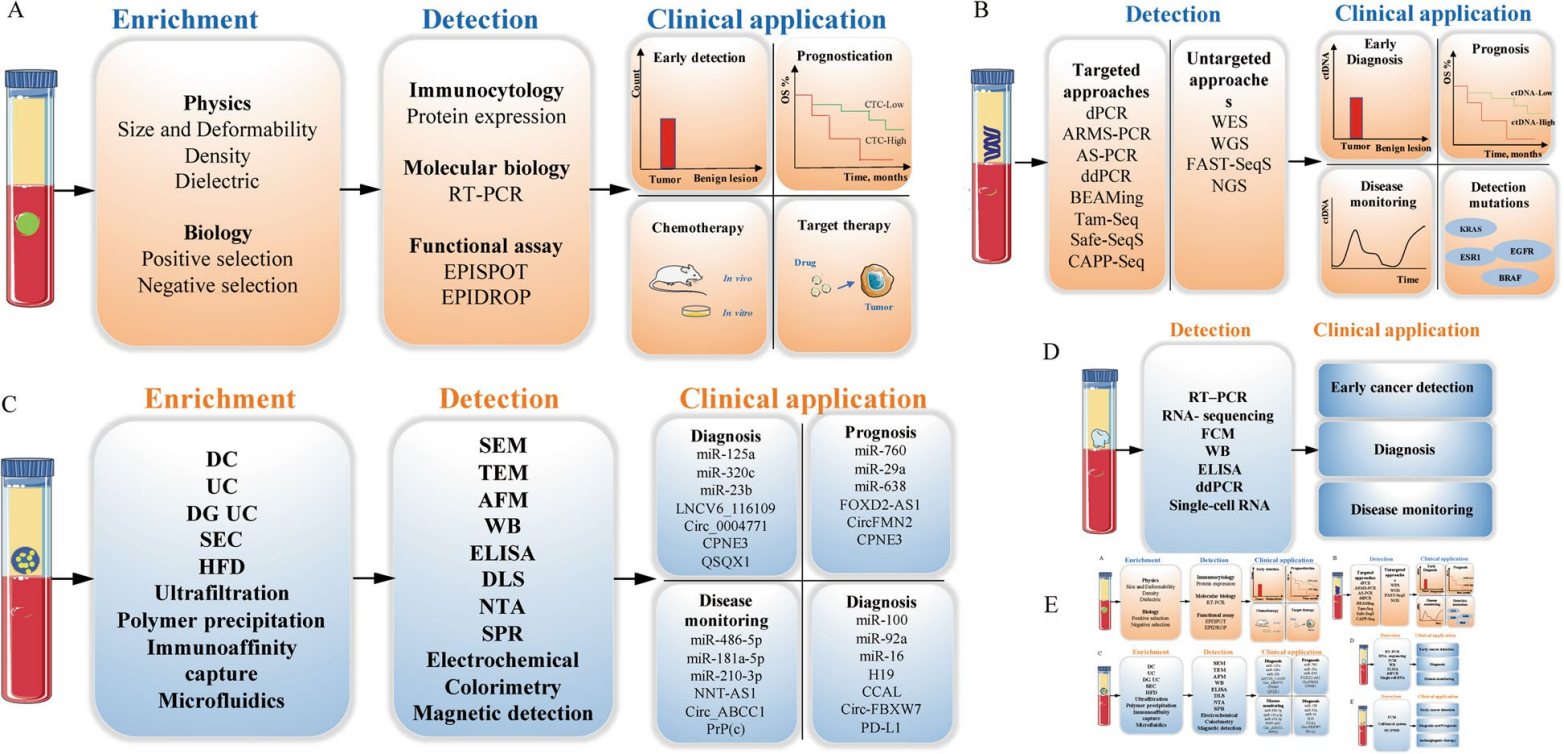
Technologies for CTC and ctDNA enrichment, detection and clinical application. **A** CTCs are preliminary enriched from whole blood sample via different enrichment techniques. Different detection technologies can help with early detection, prognostication, chemotherapy, target therapy of CRC patients. **B** ctDNA are detected from whole blood sample via targeted and untargeted approaches. ctDNA can supported with early diagnosis, prognosis, disease monitoring and detected the gene mutations of CRC patients. **C** Exosomes are enriched from whole blood sample via different enrichment techniques. Different detection technologies can help with diagnosis, prognosis, disease monitoring, therapy of CRC patients. **D** TEPs are detected from whole blood sample via different approaches. TEPs can supported with early cancer detection, diagnosis, and disease monitoring of CRC patients. **E** CTECs are detected via different approaches. CTECs can supported with early cancer detection, diagnosis and prognosis, antiangiogenic therapy of CRC patients

CTCs are cancer cells that gain access to the circulatory system and can provide cancer-related information at the DNA, RNA and protein levels, which is recognized as the fundamental reason for deadly metastatic disease in CRC and other solid tumors [29]. It seems that CTC testing has the potential to be used as a real-time “liquid biopsy” in cancer patients, which can reflect tumor detection, therapy monitoring, prognostication and individual precision therapy. However, a 1 ml blood sample often contains an extremely low concentration of CTCs, although there may be thousands of CTCs in the blood of a patient with a tumor. In addition, CTCs are highly heterogeneous, cancer-specific markers are lacking, and cell detection, enrichment and isolation remain to be further analysed. An ideal screening tool should have the advantages of reproducibility and high efficiency, as well as high sensitivity and specificity. Although technical challenges for CTC assays in CRC remain, they have begun to enter clinical trials as predictive and response biomarkers and minimal residual disease (MRD) monitoring tools (Fig. 3A).

#### CTC: detection, prognosis and progression monitoring in CRC

Early detection of CRC is critical given that patients with early-stage cancers have a better than 90% survival rate. Tsai et al. demonstrated for the first time that CTCs might be used for early cancer detection. In a prospective study, CTC detection based on the Cellmax platform showed a sensitivity of 86.9%, specificity of 97.3%, and AUC of 0.88 in CRC patients [30]. However, CTC detection is uncommon and challenging in early-stage CRC (TNM stage I–II); hence, their utility in CRC early diagnosis remains limited. Bork et al. detected that the CTC counts in early-stage CRC were as low as 9% [31]. Meanwhile, Sebastian Hinz et al. revealed that they used Ficoll extraction of mononuclear cells followed by CK20 RT–PCR to detect CTCs, with a detection rate of 30% [32]. In other investigations, CTCs were detected in almost 45% of CRC patients (TNM stage I–IV) [33, 34]. Different detection methods may have different detection rates; nevertheless, standardized methods and novel techniques will enhance CTC detection in early malignancies [35]. Much attention should be devoted to improving technical assays to accelerate the rate of CTC detection in the future. To date, CTCs have been deemed a potential noninvasive diagnostic and prognostic marker for metastatic CRC (mCRC) in multiple studies. A meta-analysis published in 2013 demonstrated that overall survival (OS) and progression-free survival (PFS) were worse in CTC-positive mCRC patients [36]. In another study, Ashton A et al. showed that CTC counts in hepatic venous > 3/7.5 mL of blood were associated with shorter DFS and OS than CTC counts in peripheral colorectal cancer liver metastasis patients (CRLM) [37]. In addition, Jia-Xing Zhao et al. also revealed that CTC detection in the initial reflux vein/portal vein blood is much more sensitive than in peripheral circulation in CRLM patients [38]. Furthermore, Fengjie Wu et al. classified CTCs into three subgroups based on the expression of EpCAM (E-CTCs), the mesenchymal cell marker vimentin (M-CTCs), or both EpCAM and vimentin (E/M-CTCs). Meanwhile, the proportions of E-CTCs, M-CTCs, E/M-CTCs and circulating tumor microemboli (CTMs) in 126 patients were 76.98, 42.06, 56.35 and 36.51%, respectively. They showed that tumor metastasis is more significantly associated with the presence of M-CTCs and CTMs than with other CTC subgroups [39]. Similarly, Yuchen Zhong et al. demonstrated that M-CTCs were correlated with tumor size, T stage, TNM stage, vascular invasion, and CEA, and M-CTCs > 1 were discovered to be associated with worse DFS [40]. According to a publication-based meta-analysis published in 2018 that included 15 published investigations with a total of 3129 patients, the presence of CTCs was highly correlated with poor survival (OS: HR = 2.36, 95% CI: 1.87–2.97; *P* = 0.006) and aggressive disease progression (PFS: HR = 1.83, 95% CI: 1.42–2.36; *P* < 0.00001). Furthermore, subgroup analyses demonstrated that CTC-positive patients also had poor OS and PFS [35]. In other words, whether CRC is early or advanced, the existence of more CTCs suggests a worse prognosis.

In the past decade, with the progression of technology and a better understanding of immune regulatory mechanisms, immune checkpoint blockers (ICBs), as potential therapeutic strategies for antitumor therapy in various cancers, have quickly evolved. Several candidate tumor-based predictive biomarkers have emerged in the treatment of IC. Microsatellite instability (MSI) and tumor mutation burden (TMB) have shown predictive value for IC inhibition [41, 42]. In regard to IC, two such immune checkpoints, cytotoxic T-lymphocyte protein 4 (CTLA-4) and programmed cell death protein 1 (PD-1), have garnered the most attention [43]. PD-1 combined with programmed cell death protein ligand 1 (PD-L1) can inhibit T cell activation and cause the immune escape of tumor cells. The expression of PD-L1 is the gold-standard biomarker for treatment selection in the field of immunotherapy. Additionally, anti-PD-1 or PD-L1 therapy has been shown to offer a good OS in several cancers [44–46]. In CRC, current immune checkpoint inhibitors (ICIs) are effective in heavily tumor mutations that are mismatch repair deficient (dMMR) or have high levels of microsatellite instability (MSI-H) [41]. However, PD-L1 biopsy may not represent the entire tumor, leading to sampling bias and abandonment of immunotherapy. Therefore, liquid biopsy might be a better way to overcome this dilemma. The evaluation of PD-L1 expression on CTCs has been assessed in CRC [47]. Recently, a study performed by Lucrezia Raimondi et al. demonstrated the expression of PD-L1 on CTCs of chemorefractory mCRC patients. In this study, they showed the usefulness of CTCs as a noninvasive real-time biopsy to evaluate PD-L1 expression in patients diagnosed with mCRC and treated with regorafenib [48]. Additionally, Arun Satelli et al. showed that CTC detection alone was not associated with poor PFS or OS in CRC, but nuclear PD-L1 expression in these patients was significantly associated with short survival durations [47].

#### CTCs: methodology and technical challenges

Since the original discovery of CTCs, multiple isolation strategies have been developed. However, due to a low concentration of CTCs in the bloodstream (approximately 1–100 cells per mL of blood) and the short half-life of CTCs in the circulation (1–2.5 h) [49], as well as the lack of cancer-specific markers, their detection remains difficult, weakening their value as a diagnostic tool [50]. Considering this dilemma, a number of platforms have been designed for detecting CTCs in blood samples. Enrichment, detection, and analysis are the three analytical procedures involved in this process. The techniques of capturing CTCs include biophysical enrichment and positive and negative immunoaffinity strategies. Diverse strategies have been introduced to enrich or extract CTCs from the blood circulation based on their physical features (size, elasticity, density and surface charge), biological properties, and expression of distinct tumor markers [51]. Racila et al. applied an antibody directed against epithelial cell adhesion molecule (EpCAM) coupled to ferrofluids and flow cytometry (FCM) to detect CTCs based on immunomagnetic CTC enrichment [52]. The CellSearch instrument, which is a widely used gold standard today, has been authorized by the Food and Drug Administration (FDA) for CTC detection in CRC. However, the EpCAM bias imposed on the enriched CTC population is a notable shortcoming of immunocapture approaches, including CellSearch [53]. Cells that have undergone epithelial to mesenchymal transition (EMT) may downregulate the epithelial marker EpCAM, making immunocapture approaches ineffective and perhaps leading to false-negative results. For this reason, AdnaTest, isolation by size of epithelial tumor cells (ISET), enzyme-linked immunosorbent spot assay (EPISPOT), fluorescence-assisted in situ hybridization (FISH), fibre-optic array scanning technology (FAST), density gradient, microfiltration, microflow, and size-dictated immunocapture chip [54] are other technologies used for CTC enrichment, detection and separation. In addition, the CTC-Chip and CTC Cluster Chip are examples of successful isolation technology combinations in the last decade. The use of microfluidic rare-cell capture technology on a larger scale in cancer patients holds much potential for uncovering significant biological characteristics of blood-borne metastases and providing a robust platform for early cancer detection and monitoring [55, 56]. Furthermore, nanotechnologies have been developed and advanced to enhance the sensitivity and specificity for the detection of CTCs. Wenzhe Li et al. reviewed the applications of nanotechnology-based liquid biopsy, bringing a fresh perspective to the clinical practice of tumor surveillance and therapy [57]. Not only can xenotransplantation into mice be used to enrich the number of CTCs in a cell culture, but it can also be utilized to facilitate further investigation [58]. To summarize, multiple ways to capture CTCs are likely to be necessary, which will facilitate future cancer research and need to be validated.

### Circulating tumor DNA in CRC 

#### ctDNA: detection, prognosis and progression monitoring in CRC

cfDNA is an emerging potential biomarker for guiding precision therapy in CRC [59, 60]. In general, cfDNA analysis is used to discover point mutations or structural variants, copy-number aberrations, microsatellite alterations, differential cfDNA length, and methylation status [61]. ctDNA arises from somatic tumor DNA fragments released into the blood circulation during cell death and has been found to contain tumor-specific molecular characteristics [62]. In a recent prospective, multicenter cohort study, plasma ctDNA was analyzed in patients with stage I to III CRC. ctDNA was detectable in 108 of 122 patients (88.5%). Furthermore, they found that ctDNA-positive patients were 7 times more likely to relapse than ctDNA-negative patients at postoperative Day 30. Similarly, ctDNA-positive patients may relapse after adjuvant chemotherapy (ACT). After regular therapy, ctDNA-positive patients were more than 40 times more likely to suffer cancer relapse during surveillance than ctDNA-negative patients (HR, 43.5; 95% CI, 9.8–193.5 *P* < 0.001). It is believed that ctDNA may be useful for risk classification, ACT surveillance, and early recurrence detection in CRC [63]. The results of this article are consistent with many previous studies [64–67]. Distant metastasis is one of the reasons for the poor prognosis of CRC. Relatively high levels of ctDNA in plasma in CRLM patients are known [68]. A single-center retrospective study was performed to investigate the impact of ctDNA on the OS of patients who underwent initial hepatectomy for resectable CRLM. In this study, they found that patients who underwent ctDNA detection before surgery had a high recurrence rate [68]. Furthermore, in another study, it was found that detectable postoperative ctDNA in resected CRLM patients had a significantly lower RFS (HR 6.3; 95% CI 2.58 to 15.2; *P* < 0.001) and OS (HR 4.2; 95% CI 1.5 to 11.8; P < 0.001) than patients with undetectable ctDNA [69]. More recently, peritoneal fluid was used to detect ctDNA for CRC peritoneal metastases (CRC-PM), and the findings suggest that ctDNA detection in peritoneal fluid may be prior to ctDNA in plasma to monitor CRC-PM [70]. Blood contains ctDNA, which can also be detected in other biological fluids, such as urine. Yu, H. et al. indicated that both plasma and urine cfDNA levels were higher in mCRC patients than in healthy individuals, which can be used to monitor disease progression in CRC patients [71].

The cfDNA methylation profile enables early diagnosis, prognosis prediction, and screening for CRC [72]. The cfDNA methylation profile enables early diagnosis, prognosis prediction, and screening for CRC [73–78]. Xianrui and colleagues discovered a novel cfDNA methylation model based on 11 methylation biomarkers to improve the detection of early-stage CRC patients in their investigation [79]. In another study, 159/267 (87%) mCRC patients showed positivity for methylated markers (e.g., EYA4, GRIA4, ITGA4, MAP3K14-AS1, MSCs) by ddPCR assays, suggesting that methylation can be used as a monitoring marker of tumor burden under different therapeutic regimens [80]. Furthermore, the promoter hypermethylation of septin 9 (SEPT9) in cfDNA has been confirmed as a potent biomarker in CRC, and the Epi proColon 2.0 kit for cell-free circulating methylated SEPT9 detection approval by the FDA as the first blood-based CRC screening test [81–83].

Almost all advanced CRC patients need further treatment after surgery, such as systemic chemotherapy, molecular targeted therapies or immunotherapies [84, 85]. In a multicenter cohort study of 96 patients with stage III colon cancer, ctDNA was detected in 15 of 88 (17%) post-chemotherapy samples. When ctDNA was detectable after chemotherapy, the estimated 3-year recurrence-free interval was 30%, compared to 77% when ctDNA was undetectable (HR, 6.8; 95% CI, 11.0–157.0; *P* < 0.001). This indicates that monitoring post-chemotherapy ctDNA can reveal information on minimal residual disease, therapeutic response, and recurrence in patients despite completion of standard adjuvant treatment [64]. In line with the aforementioned, a trial within a cohort study (MEDOCC-CrEATE) [86] and CIRCULATE-Japan clinical trials [87], confirmed that ctDNA could be utilized as a predictor of tumor recurrence and to monitor the effectiveness of adjuvant chemotherapy. A comprehensive genome-wide analysis of somatic mutations in CRC was conducted by the Cancer Genome Atlas (TCGA) network. The most commonly altered genes were APC, TP53, KRAS, PIK3CA and BRAF in CRC. In recent years, with the rapid development of next-generation sequencing (NGS), the detection of somatic mutations has become feasible for clinical application. For instance, the somatic BRAF V600E mutation seems to have a short life expectancy and is a poor indicator of response to standard chemotherapy [88]. Activated RAS mutation is the main reason for primary or secondary resistance to anti-EGFR therapy and predicts poor survival outcomes among CRC patients [89–92]. Although KRAS mutation status has been found in studies to be a biomarker for CRC response to EGFR-targeted therapy, however, a global phase III ASPECCT study detected RAS ctDNA mutations in panitumumab-treated mCRC patients, and emergent ctDNA RAS mutations were not associated with poor prognosis in panitumumab-treated patients [93]. A study carried out by E. I. Dumbrava et al. found that patients with high variant allele frequencies of PIK3CA-mutant ctDNA at baseline were associated with shorter OS [94].

In addition to CTCs, cfDNA or ctDNA, as the most important source in liquid biopsies, they have been implemented in the field of immune-oncology [95]. ctDNA detection is quantitative. Interestingly, the change in ctDNA levels during chemotherapy is related to tumor response or progression in several tumor types [96–98]. In a prospective pilot study performed by L. Cabel et al., which included NSCLC, CRC and melanoma, the PFS and OS of patients with detectable ctDNA after anti-PD-1 ICI treatment were significantly shorter than those without ctDNA detected. It provides a theoretical basis for evaluating ctDNA prior to treatment initiation [99]. In a nonrandomized, HLA-A status double-blinded study performed by Masahiro Kitahara et al., they assessed cfDNA levels in plasma by semiquantitative real-time PCR, which were collected from 93 mCRC patients (HLA-A2402 matched, *n* = 49; and HLA-unmatched, *n* = 44) prior to receiving immunochemotherapy. The PFS of patients with low cfDNA was significantly better than that of patients with high cfDNA (*P* = 0.0027). Interestingly, in the HLA-A2402-matched group, patients with low plasma cfDNA had significantly better PFS, but there was no difference in the HLA-A2402-unmatched group. This suggests that cfDNA may be a useful predictive biomarker of the outcome of immunotherapy in metastatic colorectal cancer [100]. MSI is the first cancer indication approved for ICB [41]. A recent study showed that CRC with MSI-H detected by using cfDNA-based assays was correlated with a good response to immunotherapy [101]. In addition, Tieng FYF et al. reviewed liquid biopsy-based tests to evaluate MSI in CRC [102]. TMB from cfDNA is emerging as a novel biomarker for cancer immunotherapy in several tumors [103, 104]. Le DT et al. demonstrated that CRC patients with high TMB commonly respond to PD-1/PD-L1 blockade [41]. In conclusion, the detection of ctDNA can be used for the early diagnosis of cancer, monitoring response, evaluating potential drug resistance to the treatment and prognosis (Fig. 3B).

##### ctDNA: methodology and technical challenges

Normal and tumor cells both release cfDNA into the circulation, and ctDNA is the portion of cfDNA shed by cancer cells. The investigation of ctDNA can disclose details about a cancer’s biological profile and clinical progression. According to estimates, ctDNA usually accounts for 0.01–5% of total cfDNA in patients with cancer [105]. While ctDNA has a two-hour half-life, it is cleared quickly after entering the circulation. As a corollary, ctDNA can act as a useful dynamic marker of tumor bulk and reflect therapy responses. Several technologies have emerged to detect ctDNA in recent years, including ultrasensitive targeted PCR-based approaches and next-generation sequencing (NGS) methods. The former includes digital PCR (dPCR) [106], allele-specific amplification refractory mutation system PCR (ARMS) [107], allele-specific PCR (AS-PCR) [108], droplet digital PCR (ddPCR), bead emulsification amplification and magnetics (BEAMing) [16] to detect mutations in prespecified cancer-associated mutations. And the latter such as tagged-amplicon deep sequencing (TAm-Seq) [109], Safe-Sequencing System (Safe-SeqS) [110] and personalized profiling by deep sequencing (CAPP-Seq) [111], enables simultaneous detection of the genome and multiple rare mutations in ctDNA simultaneously without the requirement of primary tumor sequencing. And the untargeted techniques, such as WGS or WES, allow for the detection of novel, clinically significant genomic aberrations without need the information about primary tumor. In general, the advantage of PCR-based methods is cost-effective and rapid, and no specific bioinformatic skills are needed. However, the main disadvantage is that they can detect a limited number of prespecified mutations. Among the PCR-based approaches, ddPCR or BEAMing can detect extremely infrequent mutations with high sensitivity; nonetheless, the DNA region assessed must be restricted [112]. Although dPCR is the most commonly used method for detecting ctDNA, the use of NGS for ctDNA detection is becoming increasingly prominent. Somatic single nucleotide variant allele frequencies (SNV VAFs), copy number aberrations (CNAs), or DNA methylation patterns are used in NGS-based methodologies to estimate ctDNA levels in plasma [113, 114]. Hangyu Zhang et al. developed an NGS-based ctDNA assay and evaluated its sensitivity and specificity while using ddPCR as a control in cetuximab-treated CRC patients. In the study, NGS actually found more mutation information than ddPCR in disease progression patients [115]. In general, NGS methods can detect a large number of mutations and analyze multiple genomic targets and alterations, but they are restricted by poorer sensitivity, higher input sample volume, and expensive and time-consuming procedures [113, 116]. In a recent meta-analysis, they compared the diagnostic accuracy of digital PCR, ARMS and NGS for detecting KRAS mutations in the cfDNA of CRC patients, and next-generation sequencing had overall high accuracy [117]. In summary, to be fit for clinical application, the ideal ctDNA assay should take into account the appropriate testing sensitivity, target scope, maximum sample throughput, and total annual expenditures.

### Exosomes in CRC

#### Exosome: detection, prognosis and progression monitoring in CRC

Exosomes are highly abundant in almost all body fluids, such as CSF, plasma, urine, amniotic fluid, and saliva [118]. Exosomes, as a means of intercellular communication, have the unique capacity to transport a diverse variety of cargo, including proteins, mRNA, miRNA, and lipid substances. There are various nucleic acids included in exosomes, such as miRNAs, lncRNAs, circRNAs, tRNAs, snRNAs and snoRNAs, which act as gene expression modulators and potential biomarkers [119–121]. In addition, exosomal proteins are generally related to membrane transport, which includes endosome-associated proteins (e.g., Rab proteins, annexins, flotillins, tumor susceptibility gene 101 protein), tetraspanins (e.g., CD9, CD63, CD81), heat shock proteins (HSP60 and HSP90) and EpCAM [122]. Compared with other types of EVs, exosomes are more promising as biomarkers and therapeutic targets because they are relatively abundant and stable in circulating entities and can transport genetic information and other biological materials. The nucleic acids and proteins found in CRC-derived exosomes are listed in Table 1. To date, there are some ongoing clinical trials of cancer immunotherapy based on exosomes for diagnostic, prognostic, predictive purposes in CRC (Table S1). In the subsequent sections, we will discuss the potential roles of exosomes as biomarkers in CRC and propose an outlook for the future direction of research in such fields (Fig. 3C).

**Table 1:** Biological functions of exosomal nucleic acids and proteins in CRC

Exosomal	Origin	Tendency	Downstream Target	Function	Reference
miR-125a-3p, miR-320c	plasma	up	NA	Early diagnostic biomarker	[123]
miR-17-92a, miR-19a	serum	up	NA	Early diagnostic biomarker	[124]]
miR-27a, miR-130a	plasma	up	NA	Early diagnostic biomarker prognostic biomarker	[125]
miR-193a-5p	plasma	down	CUX1 and ITSN1	Early diagnostic biomarker	[126]
miR-23b	plasma	down	NA	Early diagnostic biomarker	[127]
miR-99b-5p, miR-150-5p	serum	down	NA	Early diagnostic biomarker	[128]
miR-760, miR-29a, miR-92a	plasma	down	NA	Early diagnostic biomarker	[129]
miR-1229, miR-25-3p	serum	up	HIPK2/ VEGF pathway	Predict OS, Promote angiogenesis	[130]
miR-17-5p, miR-92a-3p	serum	up	NA	prognostic biomarker	[131]
miR-548c-5p	serum	down	NA	prognostic biomarker	[132]
miR-638	serum	down	NA	prognostic biomarker	[133]
miR-135a-5p	serum/plasma	up	LATS2-YAP-MMP7	promote occurrences of CRLM	[134]
miR-33a-5p, miR-210-3p	serum	down	NA	oxaliplatin sensitivity	[135]
miR-208b	serum	down	PDCD4	oxaliplatin sensitivity	[136]
miR-100, miR-92a, miR-16, miR-30e, miR-144-5p, let-7i	plasma	down	PI3K-AKT, AMPK, FoxO pathway	oxaliplatin resistance	[137]
miR-486-5p, miR-181a-5p, miR-30d-5p	plasma	down	NA	promote tumor progression	[138]
miR-210-3p	plasma	up	CELF2	promote tumor progression	[139]
LNCV6_116109, LNCV6_98390, LNCV6_38772, LNCV_108266, LNCV6_84003, LNCV6_98602.	plasma	up	NA	early diagnostic biomarker	[140]
FOXD2-AS1, NRIR, XLOC_009459	serum	up	NA	early diagnostic biomarker	[141]
NNT-AS1	serum	up	miR-496/RAP2C	diagnostic biomarker, therapeutic target	[142]
H19	serum	up	miR-141/β-catenin pathway	promote stemness, oxaliplatin chemoresistance	[143]
CCAL	serum	up	HuR/β-catenin pathway	oxaliplatin resistance	[144]
UCA1	serum	up	NA	cetuximab resistance	[145]
HOTTIP	serum	up	miR-214/ KPNA3	mitomycin resistance	[146]
circ_0004771	serum	up	NA	early diagnostic biomarker	[147]
circFMN2	serum	up	miR-1182/ hTERT pathway	promote tumor progression	[148]
circ-133	plasma	up	miR-133a/GEF-H1/RhoA	promote tumor metastasis	[149]
circPACRGL	plasma	up	miR-142-3p/miR-506-3P-TGF-1	promote proliferation and invasion	[150]
circ-ABCC1	plasma	up	β-catenin/Wnt pathway	promote tumor progression	[151]
circ-FBXW7	plasma	down	miR-128-3p	oxaliplatin sensitivity	[152]
circ_0000338	plasma	up	miR-217/miR-485-3p	5-FU resistance	[153]
ciRS-122	plasma	up	miR-122/PKM2	oxaliplatin resistance	[154]
CPNE3	plasma	up	NA	diagnostic biomarker, prognostic biomarker	[155]
QSOX1	serum	down	NA	early diagnostic biomarker	[156]
PrP(C)	serum	up	NA	therapeutic target	[157]

#### Exosomal miRNAs

MicroRNAs (miRNAs) are small and single-stranded nucleotides (approximately 20–22 nucleotides) that are regulated as oncogenes or tumor suppressors in cancer through various mechanisms [158]. Furthermore, miRNAs can also regulate the tumor microenvironment (TME), affecting tumor angiogenesis, tumor immune invasion and tumor–stromal interactions [159, 160]. Circulating miRNAs, which originate from tissue damage and cell apoptosis, may enter the bloodstream through the secretion of micro-vesicles and exosomes or bind to proteins such as HDL, LDL or AGO2 [161]. Due to the widespread availability and high specificity of exosomal microRNAs to CRC, exosomal microRNAs have been proposed as prospective target biomarkers for the diagnosis of CRC at early and advanced stages [162]. In a recent review, Katiusse Alves dos Santos et al. focused on circulating exosomal miRNAs in CRC and their role in CRC progression and therapy [163]. In the study performed by Jing Wang et al., miR-125a-3p and miR-320c were highly upregulated in plasma exosomes of patients with early-stage colon cancer. Interestingly, exosomal miR-125a-3p and exosomal miR-320c were significantly correlated with nerve infiltration but not with tumor size, infiltration depth, or differentiation degree [123]. Exosomal miR-17-92a or miR-19a overexpression in CRC patients is strongly linked to tumorigenesis and recurrence, especially in the early stages of the disease [124]. As in the above-mentioned studies, the expression of circulating exosomal miR-27a and miR-130a in plasma were significantly increased in CRC in the study by Xiangxiang Liu et al. They could be used as non-invasive indicators for CRC detection and prognosis [125]. Exosomal miRNAs may promote cancer and may also be suppressors. In a work conducted by Rui We et al., exosomal miR-193a-5p, as a tumor suppressor miRNA, was decreased significantly in CRC patients [126]. In addition, plasma miR-23b was significantly decreased in plasma samples from CRC patients and was significantly associated with clinical stage, tumor depth, distant metastasis and tumor recurrence [127]. Furthermore, according to RNA-sequence data analysis, exosomal miR-99b-5p and miR-150-5p levels were dramatically downregulated in early CRC patients compared to healthy volunteers [128]. miRNA profiling followed by validation confirmed that the levels of miR-601 and miR-760 were lower in patients with CRC or adenoma than in healthy controls. In addition, a combination of miR-760, miR-29a and miR-92a can improve the detection sensitivity for early-stage CRC [129]. The findings show that exosomal miRNAs can be used as an early indicator of CRC.

The fact that exosomal miRNAs can distinguish metastatic patients from those without is an interesting finding. Compared to nonmetastatic CRC patients, exosomal miR-1229 and miR-25-3p were more highly expressed in mCRC, as were miR-17-5p and miR-92a-3p, with high AUC values of 0.841 and 0.854 for metastatic discrimination, respectively [130, 131]. Likewise, serum exosomal miR-548c-5p and miR-638 showed lower expression levels in mCRC patients than in nonmetastatic patients [132, 133]. Furthermore, recent research conducted by Sun H et al. discovered that exosomal miR-135a-5p might be a promising target in halting CRLM. Interestingly, according to this study, hypoxia-induced exosomal miR-135a-5p correlates with the development, clinical features, and outlook of CRLM through the premetastatic niche [134]. As illustrated above, exosomal miRNAs also play an important role in the diagnosis of mCRC.

In recent years, chemoresistance has posed a major obstacle for CRC treatment, notwithstanding rapid progress in the pharmaceutical industry. Exosomal miRNAs may act as chemoresistance regulators and anticipate poor results in CRC patients. Shota Tanaka et al. found that acquired and intrinsic L-OHP-resistant CRC cells had lower expression of miR-33a-5p and/or miR-210-3p than sensitive cells, suggesting that miR-33a-5p would be a candidate biomarker of L-OHP sensitivity [135]. In another study, exosomal miR-208b was related to a decrease in oxaliplatin-based chemosensitivity in CRC, as it promotes Treg proliferation by targeting PDCD4 [136]. In a study conducted by Jiayi Han, plasma exosomal miR-100, miR-92a, miR-16, miR-30e, miR-144-5p, and let-7i could significantly distinguish chemoresistant patients from chemo-sensitive patients, and these miRNAs were closely linked with RNA polymerase II transcription and enriched in the PI3K-AKT, AMPK, and FoxO signaling pathways [137]. A systematic review comprehensive explanation of the role of exosomal miRNA in colorectal cancer chemotherapy resistance. According to this review, exosomal miRNAs, including miR-21, miR-92a-3p, miR-196-5p, miR-19b, and miR204-5p, increase chemoresistance, while exosomal miRNAs, including miR-128-3p, miR-1229-5p, miR-96-5p, miR-1246, miR-21-5p, miR-425, miR-135b, and miR-46,146, enhance drug sensitivity. By effectively regulating exosomal miRNAs, it can successfully overcome drug resistance and improve cancer treatment efficacy [164].

Exosomes are regarded as crucial mediators of the heterogeneity of the TME, and the occurrence and development of CRC may be influenced by the TME. Moreover, the hypoxic TME serves as a master regulator in each stage of tumor development. Exosomal oxygen-sensitive miRNAs 486-5p, 181a-5p and 30d-5p are considered circulating markers of tumor hypoxia in CRC patients [138]. In addition, under hypoxic conditions, CRC cells can secrete exosomal miR-210-3p to remodel the TME by suppressing CELF2 expression to boost tumor growth [139]. These findings shed light on the mechanism of colorectal cancer progression as well as prospective therapeutic targets for CRC.

Therefore, exosomal miRNAs can be considered promising biomarkers in the development and progression of CRC, providing valuable information on early and advanced diagnosis, prognosis, response to therapy and prediction of treatment. However, further validation is needed to ensure the reproducibility of the results.

#### Exosomal lncRNAs

LncRNAs are ncRNAs with a length of longer than 200 nucleotides that do not encode proteins. According to their function in tumors, lncRNAs can be divided into two classes: oncogenic lncRNAs and tumor-suppressor lncRNAs. Importantly, lncRNAs are relatively stable and can detect cancer-related lncRNAs in blood or other body fluids. LncRNAs detected in serum or plasma could be used as potential biomarkers in different types of tumors. However, there are few reports on the expression of exosomal lncRNAs as potential noninvasive diagnostic biomarkers in CRC. In recent years, more studies have demonstrated that circulating lncRNAs, such as HOX transcript antisense RNAs (HOTAIR), colon cancer-associated transcripts (CCAT), metastasis-associated lung adenocarcinoma transcript 1 (MALAT-1), hepatocellular carcinoma upregulated lncRNA (HULC), HOXA transcript at the distal tip (HOTTIP) and H19, have been confirmed to be associated with CRC development, invasion and metastasis and present as noninvasive molecular markers [165–167]. Hu et al. investigated plasma exosomal lncRNAs in CRC patients compared to healthy controls, discovering LNCV6_116109, LNCV6_98390, LNCV6_38772, LNCV_108266, LNCV6_84003, and LNCV6_98602, which were upregulated in CRC patients [140]. Miao Yu et al. showed that exosomal FOXD2-AS1, NRIR and XLOC_009459 levels were significantly upregulated in 203 CRC patients and 80 early-stage CRC patients compared to 201 healthy individuals, possessing AUCs of 0.728, 0.660, and 0.682 for CRC [141]. In another study, the serum levels of nucleotide transhydrogenase antisense RNA 1 (NNT-AS1) were significantly upregulated in patients with CRC compared with healthy donors. Interestingly, NNT-AS1 levels were significantly decreased after surgery [142]. According to the findings, exosomal lncRNAs have the potential to be used as diagnostic biomarkers for CRC, including early-stage CRC.

Recently, some studies have demonstrated that exosomal lncRNAs mediate cell-to-cell communication within the TME, contributing to cancer cell progression and chemoresistance. For example, upregulation of CAF-derived exosomal H19 can promote the stemness of CSCs and enhance chemoresistance by activating the Wnt pathway [143]. In addition, it has been shown that exosomal colorectal cancer-associated lncRNA (CCAL) directly binds with mRNA stabilizing protein human antigen R (HuR) and promotes the resistance of CRC cells to oxaliplatin [144]. Therefore, it is worth looking into the novel lncRNAs enriched in exosomes derived from endothelial cells surrounding the TME, which may play an essential role in tumor progression. Yang et al. reported that exosomal lncRNA-UCA1 can transmit cetuximab resistance to sensitive cells, and its expression is closely related to the clinical status of cetuximab therapy in CRC patients [145]. Interestingly, Chen et al. revealed that exosomal HOTTIP is highly expressed in mitomycin-resistant CRC cells and can increase the resistance of CRC to mitomycin by impairing miR-214-mediated degradation of KPNA3 [146]. Targeting exosomal lncRNAs might be a promising option for addressing chemoresistance in CRC.

Therefore, exosomal lncRNAs can be considered promising biomarkers in early and advanced diagnosis, prognosis, response to therapy and prediction of treatment. In summary, further studies need to be carried out to ensure the clinical applicability of exosomal lncRNAs.

#### Exosomal circRNAs

The main length of circRNAs in exosomes is 200–600 bp. CircRNAs are a subset of endogenous noncoding RNAs with a covalently closed continuous loop that are more stable and have longer half-lives [168–170]. They have several functions, including acting as miRNA sponges and interacting with RNA-binding proteins, to regulate transcription and alternative splicing [171]. Extensive studies have revealed that dysregulated circRNAs are involved in the development of a variety of malignancies. Exosomal hsa_circ_0004771 was significantly upregulated in patients with colorectal cancer and was downregulated in the serum of postoperative CRC patients, implying that circulating hsa_circ_0004771 might be used as an early diagnostic biomarker for CRC [147]. In a recent study, Li et al. demonstrated that circumformin 2 (circFMN2) is upregulated in CRC plasma, and it may boost CRC proliferation by directly binding with miR-1182 and enhance human telomerase reverse transcriptase [148]. Another study proved that hypoxia-induced exosomal circ-133 is upregulated in CRC patients and promotes tumor metastasis by targeting the GEF-H1/RhoA axis [149]. In addition, Shang et al. discovered that in CRC patients, circPACRGL expression was markedly increased. Moreover, the study suggested that circPACRGL can accelerate CRC cell proliferation and invasion, along with neutrophil differentiation from N1 to N2 [150].

Over the last decade, circRNAs have been reported as important mediators related to the development of chemoresistance in various tumors, such as gastric cancer [172, 173], non-small-cell lung cancer [174], esophageal cancer [175], pancreatic cancer [176, 177], colorectal cancer [152–154, 178], glioma [179, 180], and osteosarcoma [181, 182]. However, there are few studies on exosomal circRNA drug resistance to tumors, and the mechanism remains unknown. Recent studies have demonstrated that exosomal circRNAs play important roles in CRC chemoresistance. For example, exosomal circRNAs such as hsa_circ_0000677 (circ-ABCC1) promote the stemness and sphere formation of CRC cells, along with tumor invasion and progression via the Wnt pathway [151]. In addition, a study by Yeqing Xu et al. confirmed that circ-FBXW7 delivery by exosomes could ameliorate chemoresistance to oxaliplatin in CRC by directly interacting with miR-128-3p, suggesting a feasible therapeutic option for oxaliplatin-resistant CRC patients [152]. Another study verified that exosomal circ_0000338 can enhance 5-FU resistance in CRC by negatively regulating miR-217 and miR-485-3p, indicating a promising diagnostic and therapeutic marker for 5-FU-based chemotherapy in CRC patients [153]. Interestingly, exosomal ciRS-122 derived from chemoresistant CRC cells could promote glycolysis and chemoresistance to oxaliplatin by regulating the ciRS-122–miR-122–PKM2 pathway [154]. The above research suggests a potential novel therapeutic target and establishes a foundation for future clinical applications in drug-resistant CRC.

#### Exosomal proteins

In recent years, with the development of proteomics, the role of exosomal proteins in predicting tumor occurrence, diagnosis and therapy has received much attention. Many studies have shown that the expression of exosomal proteins in different tumors varies greatly [183–185]. Bo Sun et al. demonstrated that CRC patients with lower exosomal CPNE3 levels had substantially better DFS and OS than those with higher exosomal CPNE3 levels [155]. Nicole Gang et al. investigated activation status-related exosomal proteins in CRC. Proteomic analyses revealed that the exosomal-associated protein QSOX1 was significantly reduced in plasma exosomes from CRC patients [156]. In another study, exosomal cellular prion protein (PrP) was overexpressed in CRC under a hypoxic TME. They regulate CRC cell behavior and tumor progression. Furthermore, the application of an anti-PrP antibody with 5-fluorouracil significantly suppressed CRC progression in vivo. Taken together, these findings suggest that the coadministration of anti-PrP antibody and anticancer drugs may be an effective therapeutic strategy in colorectal cancer [157].

Additionally, cancer cells can secrete higher concentrations of exosomal PD-L1 rather than present PD-L1 on their cell surface in different tumors. Therefore, exosomal PD-L1 might serve as a worse prognosis in several types of tumors [186]. Exosomal PD-L1 has significant implications for immunotherapeutic approaches to cancer therapy in CRC [187].

#### Exosomes: methodology and technical challenges

The lack of standardized protocols for specimen preparation, exosome isolation and analysis, which could affect reproducibility, is a limitation to the clinical application of exosomes. Exosome isolation procedures are based on physical (density and size) and biological characteristics, similar to CTCs [188]. Because exosomes are commonly mixed with other biofluid components, so effective isolation is the key step for experimental research. Thus far, the commonly used exosome isolation strategies are classified as density-based, size-based, surface components-based, and precipitation methods [189, 190]. Traditional exosome isolation can be performed using a variety of methods, including differential centrifugation (DC), ultracentrifugation (UC), density gradient ultracentrifugation (DG UC), ultrafiltration, size-exclusion chromatography (SEC), hydrostatic filtration dialysis (HFD), polymer precipitation, and immunoaffinity capture. The gold standard approach for isolating exosomes is UC [191]. It is determined by the size of particles and the solution’s viscosity. While the approach is simple and straightforward, it still has a low recovery efficiency and a low purity level [192]. One of the prominent size-based exosome isolation techniques is ultrafiltration. The method has the advantages of a simpler and faster procedure, a higher yield, and no need for special equipment. However, the exosomes recovery rate may be low due to the deformation and breaking up of exosomes [193]. SEC, another size-based methodology that is superior to ultracentrifugation, provides better exosome recovery [194]. In 2014, Musante et al. used HFD method to successfully isolate exosomes from urine samples. And the method was proved to be simple, quick, and efficient [195]. Based on the chemical properties of exosomes, the polymer-based precipitation separation method is used to precipitate exosomes. To date, the polyethylene glycol (PEG) approach is the most widely utilized polymer-based exosome separation method. The method is straightforward to use, requires no special equipment, and can scale up to huge sample volumes. The microplate-based immunocapture technique and the immunoaffinity capture/magneto-immunocapture.

based on immunoaffinity were proposed for immunological exosome separation to capture exosomes [196, 197]. The various proteins on the membrane of exosomes are ideal biomarkers for immunological separation of exosomes. Furthermore, it is easy to gather high purity and isolate a certain subtype of exosomes by targeting specific proteins. Advances in microfluidics, including acoustic fluid separation [198], dielectrophoretic (DEP) separation [199], and deterministic lateral displacement (DLD) separation [200], have played an increasingly important role in exosome separation and detection. Together with isolation methods, exosome detection methods include scanning electron microscopy (SEM), transmission electron microscopy (TEM), atomic force microscopy (AFM), enzyme-linked immunosorbent assay (ELISA), dynamic light scattering (DLS), nanoparticle tracking analysis (NTA), western blot (WB), colorimetry, surface plasmon resonance (SPR), magnetic detection, electrochemical detection, and so on [201–204]. In summary, standardized protocols for specimen preparation, exosome isolation, and analysis should be taken into consideration to be suited for clinical utilization.

### TEPs in CRC 

#### TEPS: detection, prognosis and progression monitoring in CRC

Platelets are small (2–4 μm), circulating anucleate hematopoietic cells generated from MKs that subsequently mature and even divide in the bloodstream [205]. Platelets are the second most abundant cell type in peripheral blood and have been described as having an important role in haemostasis and thrombosis [23]. TEPs are an emerging concept that have attracted significant attention for their potential use in cancer diagnostics. Cancer cells can activate platelets directly by their adhesion to circulating platelets and indirectly by numerous released factors [206]. The direct interaction of platelets with cancer cells appears to be necessary for cancer progression. Peterson et al. demonstrated that VEGF, PDGF, and platelet Factor 4 (PF4) are elevated in platelets of CRC patients [207]. On the one hand, cancer cells can also “educate” platelets through both direct and indirect mechanisms stimulating tumor cell-induced platelet aggregation (TCIPA) by delivering their cargo, including RNA and protein profiles [208]. However, the role of TEPs in RNA regulation may alter their spliced RNA profile [209, 210]. TCIPA is considered to promote angiogenesis and metastasis in tumors, so it is negatively correlated with prognosis and survival [211]. An initial work by Best et al. distinguished cancer patients from healthy individuals by using RNA-seq analysis. Furthermore, mutant oncogenic drivers, including KRAS-, EGFR-, PIK3C4-, MET- and HER-2-positive tumors, were precisely distinguished, suggesting that TEPs may provide precise tools for cancer diagnosis and targeted therapies [28]. Additionally, several studies have reported that the platelet RNA profile changes in several cancer types, including non-small-cell lung cancer (NSCLC) [212–216], hepatocellular carcinoma (HCC) [217–219], glioma [220, 221] and breast carcinomas [222]. However, the role and function of TEP mRNAs in CRC are still not clear. Recent studies have demonstrated that TEPs play essential roles in tumor progression and metastasis [223, 224]. Liu Yang et al. proved that TIMP1 mRNA levels are higher in platelets from CRC patients than in platelets from healthy volunteers, suggesting that it may be used as a diagnostic biomarker for CRC [225]. Moreover, a study by Qian et al. showed that TEPs and platelet counts are associated with the prognosis of resectable lung cancers and CRC [226].

During previous decades, many studies have explored the protein profiles of platelets. However, not all mRNAs in platelets are translated into proteins. Londin et al. showed that only 40% of platelet mRNAs are translated into corresponding proteins [227]. To date, researchers have only focused on the transfer of tumor-derived RNA into platelets but not on its associations with platelet protein profiles [27, 227]. Despite their anucleate nature and short lifespan, platelets are becoming well recognized. Importantly, the protein and RNA profiles of TEPs are dynamically transformed in various types of cancer patients. TEPs are considered mediators that influence several hallmarks of cancer, such as immune system evasion and the TME. Increasing evidence suggests that TEPs can influence the development of tumor. The easy accessibility and separation of TEPs makes them more advantageous than CTCs, ctDNA and exosomes in the diagnosis of cancer. When looking for novel approaches to deal with malignant tumor, TEPs can be used as a promising tool for diagnosis, prognosis and treatment (Fig. 3D).

##### TEPs: methodology and technical challenges

Currently, the focus of most research on TEPs is changes in RNA content and modification of the proteome. Additionally, technologies have emerged to detect TEPs, including conventional RT–PCR, RNA sequencing, FCM analysis, WB and ELISA approaches [207, 209, 220, 221]. New methods, such as ddPCR and single-cell RNA sequencing, can assess tumor RNAs in plasma with enhanced accuracy and sensitivity to improve the detection of mutant RNAs within TEPs [228, 229]. TEP detection is expected to increase as a result of these advancements, which will aid future cancer research. In summary, there are still few studies on TEPs in CRC, and the detection methods need to be continuously improved in the future.

### Other Liquid Biopsy Components in CRC

TME components are also a minimally invasive source of liquid biopsy and are rapidly becoming the focus of precision medicine research [230]. In recent studies, researchers discovered new types of circulating non-tumoral cells, as well as their derived markers and extracellular matrix components, which have clinical utility for diagnosis, prognosis, and therapeutic response [231]. Circulating endothelial cells (CECs) are vascular endothelial cells found in the blood that act as surrogate markers of endothelial damage and an increase in a variety of disorders such as vascular, autoimmune, infectious, and tumor [232–235]. CECs, which are derived from mature endothelial cells and endothelial progenitor cells of the existing vessel wall as well as endothelial progenitor cells from bone marrow (EPCs), proliferate dramatically during tumor progression and play an important role in tumor angiogenesis [236]. Several research suggests that circulating tumor-derived endothelial cells (CTECs) may play a prognostic role in CRC, with a higher prognostic value than CTCs [237, 238]. In a recent study, CTECs may become a diagnostic method in early-stage (≤IIA) CRC patients. At the time of diagnosis, during treatment, and throughout the course of the disease, CTECs may provide important information about the underlying tumor vasculature [239]. Interestingly, several studies show that CTECs have predictive value in bevacizumab-treated mCRC patients [240, 241]. Matsusaka et al. discovered that CXCR4-positive CEC were significantly associated with longer PFS and OS as compared other indicators investigated [242]. Similarly, Gootjes et al. observed that CECs and CD276-psoitive CTECs based on FCM were significantly increased after treatment with bevacizumab plus chemotherapy in mCRC patients [243]. To analyze CECs and their subpopulations, two analytical techniques are available: multiparameter FCM and the CellSearch system [237]. Subtraction enrichment-immunofluorescence in situ hybridization (SE-iFISH) is a novel strategy for detecting CTCs and CTECs with high sensitivity in order to identify malignant nodules [244]. Much more research is needed to determine the clinical utility of CECs and CTECs as biomarkers of antiangiogenic therapy in CRC patients (Fig. 3E).

Circulating immune cells serve as a noninvasive indicator of immunotherapy responsiveness in CRC. A study has shown that circulating T cell lymphocyte subsets are also confirmed as mCRC biomarkers [245]. The result showed that the decrease of CD4+ and Treg ratios had a better OS during the folinic acid, 5-FU and irinotecan (FOLFIRI) plus bevacizumab treatment. Additionally, A low proportion of circulating Tregs among CD4^+^ cells and a high CD8: Treg ratio have prognostic value following VEGF-targeting therapy [246]. Interestingly, the systemic immune-inflammation index, the ratios of different immune cells and immune cells to platelets are also prognostic and predictive biomarkers in mCRC patients, including platelet-to-lymphocyte ratio (PLR), neutrophil-lymphocyte ratio (NLR) [247–249]. Other TME components, such as extracellular matrix (ECM) components and collagen fragments from ECM have been studied as diagnostic and prognostic biomarkers in many tumors, but their role in mCRC is unknown [250, 251].

## Future perspectives and conclusion

Within the past decade, the field of liquid biopsy has grown rapidly. Liquid biopsy is a valid alternative to tissue re-biopsy. In general, liquid biopsy is noninvasive, overcomes tumor heterogeneity and can allow real-time monitoring of tumor progression, recurrence or therapeutic response [252]. There are also ongoing clinical trials from the US National Laboratory of Medicine (NIH) for liquid biopsy in CRC, aiming at predicting which patients require special monitoring and individualized therapy. Liquid biopsy opens a new avenue for CRC early detection, disease monitoring, treatment response and therapeutic resistance. In the present review, we summarized the techniques currently applied to liquid biopsy and described the different circulating biomarkers in body fluids and their clinical potential for precision therapy of CRC.

However, on its own, each approach has limitations. Indeed, there are still several technical factors clearly hindering the potential translation of liquid biopsy biomarkers into clinical practice. First and foremost, CTCs and cfDNA collected from CRC patients are commonly poorly concentrated [253, 254]. Second, there is a lack of standard methodology of isolation, enrichment or detection. Therefore, applying different technologies or assays to detect CTCs or ctDNA may lead to diverse sensitivities and specificities [255, 256]. Last, there is an urgent need for more multicentre, larger, longer-term studies to achieve the clinical use of liquid biopsies, including clinical trials.

In recent years, an increasing number of studies have focused on exosomes and their roles in cancer progression. Exosomes are involved in many processes of tumor initiation, development and metastasis, including EMT, tumor angiogenesis, extracellular matrix remodelling, organ-specific metastasis, and immune evasion. Exosomes can also contribute to drug resistance in cancers. Because exosomes are easier to isolate than CTCs and cfDNA in tumors, an increasing number of studies will be focused on exosomes in the diagnosis of cancers at an early stage in the future. However, there are still some problems in clinical application, such as low targeting efficiency and easy phagocytosis by the immune system. Moreover, the method of isolation and purification of exosomes is wasting time and energy. Therefore, more research should be done to solve these problems and develop more effective clinical applications of exosomes. The advantages of TEPs are due to their considerable abundance and ease of isolation in the blood, their high-quality RNA, and their ability to process RNA in response to external signals [209, 257, 258]. Combinatorial analysis of TEP RNA with complementary biosources such as exosomes, ctDNA, and CTCs will enhance the detection of cancer in an early stage and promote noninvasive disease surveillance. The next frontier for liquid biopsies is whether they might act as predictive biomarkers for immunotherapy. TMB in ctDNA and IC protein in CTCs play important roles in tumor immunotherapy. However, due to insufficiently and incompletely understood molecular mechanisms, liquid biopsy has not yet been implemented in immuno-oncology in the clinic, but promising data and rapidly advancing technologies suggest that this approach has the potential to personalize the clinical management of cancer patients receiving ICIs. In addition, more research is needed to prove this hypothesis, especially in clinical trials.

The universal replacement of tumor biopsies with liquid biopsies seems unrealistic; however, as ctDNA, CTCs, exosomes, TEPs and additional blood tests improve, it seems likely that they will become an increasingly used tool for CRC in early detection, postoperative monitoring, treatment response and therapeutic resistance. In summary, liquid biopsy is a key part of precise medicine and is believed to be a clinical reality in the near future.

## Supplementary Information

**Additional file 1.**

## Data Availability

Not applicable.

## Abbreviations

CRC: colorectal cancer
CTCs: circulating tumor cells
ctDNA: circulating tumor DNA
TEPs: tumor-educated platelets
CTECs: circulating tumor-derived endothelial cells
CSF: cerebrospinal fluid
cfDNA: circulating free DNA
WES: whole exome sequencing
WGS: whole genome sequencing
NCCN: National Comprehensive Cancer Network
EVs: extracellular vesicles
MKs: megakaryocytes
MRD: minimal residual disease
OS: overall survival
PFS: progression free survival
CRLM: colorectal cancer liver metastases
CTMs: circulating tumor microemboli
ICB: immune checkpoint blockers
MSI: microsatellite instability
TMB: tumor mutation burden
CTLA-4: Cytotoxic T-lymphocyte protein 4
PD-1: programmed cell death protein 1
PD-L1: programmed cell death protein ligand 1
ICIs: immune checkpoint inhibitors
dMMR: mismatch repair deficient
EpCAM: epithelial cell adhesion molecule
FCM: flow cytometry
FDA: Food and Drug Administration
EMT: epithelial to mesenchymal transition
ISET: isolation by size of epithelial tumor cells
EPISPOT: enzyme-linked immunosorbent spot assay
FISH: fluorescence-assisted in situ hybridization
FAST: fiber-optic array scanning technology
ACT: adjuvant chemotherapy
SEPT9: septin 9
NGS: Next generation sequencing
dPCR: digital PCR
ARMS: allele-specific amplification refractory mutation system PCR
AS-PCR: allele-specific PCR
ddPCR: droplet digital PCR
BEAMing: bead emulsification amplification and magnetics
TAm-Seq: tagged-amplicon deep sequencing
Safe-SeqS: Safe-Sequencing System
CAPP-Seq: personalized profiling by deep sequencing
SNV VAFs: Somatic single nucleotide variant allele frequencies
CNAs: copy number aberrations
TME: tumor microenvironment
HOTAIR: HOX transcript antisense RNAs
CCAT: colon cancer-associated transcripts
MALAT-1: metastasis-associated lung adenocarcinoma transcript 1
HULC: hepatocellular carcinoma upregulated lncRNA
HOTTIP: HOXA transcript at the distal tip
NNT-AS1: nucleotide transhydrogenase antisense RNA 1
CCAL: colorectal cancer associated lncRNA
HuR: human antigen R
circFMN2: circumformin 2
DC: differential centrifugation
UC: ultracentrifugation
DG UC: density gradient ultracentrifugation
SEC: Size-exclusion Chromatography
HFD: hydrostatic filtration dialysis
PEG: polyethylene glycol
DEP: dielectrophoretic
DLD: deterministic lateral displacement
SEM: scanning electron microscopy
TEM: transmission electron microscopy
AFM: atomic force microscopy
ELISA: enzyme-linked immunosorbent assay
DLS: dynamic light scattering
NTA: nanoparticle tracking analysis
WB: western blot
SPR: surface plasmon resonance
TCGA: the Cancer Genome Atlas
PF4: platelet factor 4
TCIPA: tumor cell induced platelet aggregation
NSCLC: non-small cell lung cancer
HCC: hepatocellular carcinoma
CECs: circulating endothelial cells
EPCs: endothelial progenitor cells
SE-iFISH: Subtraction enrichment-immunofluorescence in situ hybridization
PLR: platelet-to-lymphocyte ratio
NLR: neutrophil-lymphocyte ratio
ECM: extracellular matrix
NIH: National Laboratory of Medicine
