# Supplementary material for: Liquid biopsy at the frontier of detection, prognosis and progression monitoring in colorectal cancer
Source: Mol Cancer. 2022 Mar 25;21:86. doi: 10.1186/s12943-022-01556-2 (PMC8951719; doi:10.1186/s12943-022-01556-2)
Supplement: Supplementary file 1 — Additional file 1. [file 12943_2022_1556_MOESM1_ESM.docx]

Supplement Table 1. liquid biopsy−guided ongoing clinical trials in CRC.

| Trial Name | Status | Sample Size | Study Type | Conditions | Intervention | Detection Assay |
| --- | --- | --- | --- | --- | --- | --- |
| POACC-1  (NCT03700411)  Czech | Recruiting | 120 | Interventional | CTC  CRC | Drug: Morphine, Piritramid, Epidural | NA |
| POACC-2  (NCT03700541)  Czech | Recruiting | 200 | Interventional | CTC  CRC | Drug: Morphine,  Piritramid | NA |
| IMMC-06  (NCT00133913)  US | Unknown | 486 | Observational | CTC  mCRC | NA | NA |
| (NCT01596790)  France | Completed | 168 | Interventional | CTC  mCRC | NA | EPISPOT, Cellsearch |
| COL016  (NCT01163305)  China | Completed | 84 | Interventional | CTC  mCRC | Adjuvant ChT | NA |
| VISNU-2  (NCT01640444)  Span | Completed | 240 | Interventional | CTC  mCRC | Adjuvant ChT | NA |
| (NCT02005913)  China | Unknown | 600 | Observational | CTC  CRC | NA | NA |
| (NCT03295591)  China | Unknown | 77 | Observational | CTC  mCRC | NA | NA |
| ZSCTC3  (NCT04917289)  China | Not yet recruiting | 100 | Interventional | CTC  CRC | Adjuvant ChT  Radiography | NA |
| ZSCTC2  (NCT04917276)  China | Not yet recruiting | 100 | Observational | CTC  Stage IV CRC | NA | NA |
| ADNCHIR  (NCT03809403)  France | Unknown | 40 | Observational | CTC  CRC | NA | NA |
| 39748EP  (NCT01722903)  USA | Completed | 25 | Observational | CTC  Stage IV CRC | NA | NA |
| CMx-CTC-CRC-001  (NCT03476122)  China | Unknown | 750 | Observational | CTC  CRC | NA | NA |
| (NCT02450422)  China | Completed | 60 | Observational | CTC  CRC | NA | FCM,  RT-PCR |
| (NCT02948985)  China | Unknown | 100 | Observational | CTC  mCRC | Adjuvant ChT | NA |
| (NCT02029326)  Korea | Completed | 30 | Observational | CTC  CRC | NA | NA |
| ACABC  (NCT02602938)  China | Unknown | 40 | Interventional | CTC  CRC | Drug: Aspirin | NA |
| (NCT03640572)  Poland | Completed | 91 | Observational | DTC  CRC | NA | NA |
| (NCT02186236)  USA | Completed | 84 | Observational | CTC  CRC | NA | NA |
| (NCT01189903)  Singapore | Unknown | NA | Interventional | CTC  CRC | Adjuvant ChT | NA |
| (NCT01828918)  China | Unknown | 300 | Interventional | CTC  CRC | miRNAs | NA |
| (NCT03008499)  China | Completed | 20 | Interventional | CTC  mCRC | NA | NA |
| (NCT01196130)  USA | Not yet recruiting | 1275 | Observational | CTC  CRC | NA | NA |
| (NCT03357276)  China | Completed | 30 | Interventional | CTC  CRC | Mix vaccine | NA |
| VISNU-1  (NCT01640405)  Spain | Completed | 350 | Interventional | CTC  CRC | Adjuvant ChT | NA |
| ARFIM  (NCT03960021)  France | Recruiting | 20 | Interventional | CTC  mCRC | Radiography | NA |
| (NCT01919151)  Norway | Unknown | 500 | Observational | CTC  CRC | NA | NA |
| EXACT  (NCT03975491)  USA | Recruiting | 60 | Interventional | CTC  CRC | NA | NA |
| CRC  (NCT04513431) | Not yet recruiting | 18 | Interventional | CTC  mCRC | Anti-CEA-CART | NA |
| (NCT03031691)  USA | Completed | 7 | Interventional | CTC  mCRC | Adjuvant ChT | NA |
| (NCT02080650)  USA | Completed | 62 | Interventional | CTC  CRC, other tumors | NA | c-MET  EpCAM |
| (NCT03551951)  USA | Recruiting | 320 | Observational | CTC  CRC, other tumors | NA | NA |
| (NCT03193710)  China | Recruiting | 260 | Interventional | CTC  mCRC | Adjuvant ChT | NA |
| (NCT01167725)  USA | Unknown | 340 | Interventional | CTC  CRC | Adjuvant ChT | NA |
| (NCT00924092)  USA | Completed | 25 | Interventional | CTC  CRC, other tumors | GI-6207,  Yeast CEA Vaccine | NA |
| (NCT00560560)  USA | Completed | 168 | Interventional | CTC  CRC | CP-751,871 | NA |
| (NCT04279509)  Singapore | Recruiting | 35 | Interventional | CTC  CRC, other tumors | NA | NA |
| (NCT00483080)  Italy | Completed | 46 | Interventional | CTC  CRC | Drug: NGR-hTNF | NA |
| (NCT02983578)  USA | Active, not recruiting | 53 | Interventional | CTC  CRC, other tumors | Adjuvant ChT | NA |
| (NCT00335595)  Spain | Completed | 480 | Interventional | CTC  CRC | Adjuvant ChT | NA |
| (NCT037844620)  USA | Recruiting | 100 | Interventional | CTC  CRC | NA | NA |
| (NCT02838836)  USA | Recruiting | 120 | Observational | CTC  CRC, other tumors | NA | NA |
| COCA-MACS  (NCT02872779)  France | Unknown | 74 | Interventional | ctDNA  CRC | NA | NA |
| CANSIDATE  (NCT04704960)  Korea | Not yet recruiting | 400 | Observational | ctDNA  CRC | NA | NA |
| CITCCA  (NCT04726800)  Sweden | Recruiting | 300 | Observational | ctDNA  CRC | NA | NA |
| CIRCUS  (NCT04186117)  France | Recruiting | 100 | Interventional | CTC  CRC | NA | NA |
| (NCT02869269)  Korea | Completed | 67 | Observational | ctDNA  CRC | NA | NA |
| ALGECOLS  (NCT01198743)  France | Completed | 261 | Observational | ctDNA  CRC | NA | NA |
| CTC  (NCT02556281)  France | Unknown | 216 | Interventional | ctDNA  CRC | NA | NA |
| (NCT04104633)  France | Recruiting | 20 | Interventional | ctDNA  CRC | NA | NA |
| (NCT01983098)  France | Unknown | 100 | Observational | ctDNA  CRC | NA | NA |
| TRACC  (NCT04050345)  UK | Recruiting | 1000 | Observational | ctDNA  Stage Ⅰ-III CRC | Observational | 16-plex  PCR/NGS |
| STUDY19070371  (NCT02842203)  USA | Active, not recruiting | 137 | Observational | ctDNA  Stage III CRC | NA | NA |
| FZ-CON-001  (NCT05131243)  China | Not yet recruiting | 100 | Interventional | ctDNA  Stage Ⅱ-III CRC | Adjuvant ChT | Safe-SeqS |
| (NCT04786600)  USA | Recruiting | 78 | Interventional | ctDNA  mCRC | FDA-approved drugs | NA |
| (NCT05051592)  Singapore | Recruiting | 40 | Observational | ctDNA  CRC | NA | NA |
| IMPROVE  (NCT03637686)  Denmark | Recruiting | 1800 | Observational | ctDNA  CRC | NA | ddPCR |
| (NCT04752930)  China | Recruiting | 138 | Interventional | ctDNA  CRC | NA | NA |
| (NCT03737591)  China | Unknown | 500 | Observational | ctDNA  CRC | NA | NGS |
| (NCT03416478)  China | Unknown | 50 | Observational | ctDNA  CRC | NA | NGS |
| (NCT03416478)  China | Unknown | 50 | Observational | ctDNA  CRC | NA | NGS |
| (NCT04486378)  USA | Recruiting | 201 | Interventional | ctDNA  CRC | Drug: RO7198457 intravenous | NA |
| (NCT03312374)  China | Unknown | 350 | Observational | ctDNA  CRC | NA | NA |
| (NCT04264702)  USA | Recruiting | 2000 | Observational | ctDNA  CRC | NA | NA |
| (NCT04813627)  USA | Recruiting | 200 | Observational | ctDNA  Stage Ⅱ-III CRC | NA | NA |
| (NCT03737539)  China | Recruiting | 300 | Observational | ctDNA  CRC | NA | NA |
| (NCT04831528)  China | Not yet recruiting | 100 | Interventional | ctDNA  mCRC | Adjuvant ChT | NA |
| RASINTRO  (NCT03259009)  France | Unknown | 73 | Interventional | ctDNA  mCRC | NA | NA |
| ERASE-TMZ  (NCT05031975)  Italy | Not yet recruiting | 35 | Interventional | ctDNA  mCRC | Adjuvant ChT | NA |
| IMPROVE-IT2  (NCT04084249)  Denmark | Recruiting | 254 | Interventional | ctDNA  CRC | NA | NGS |
| (NCT03828396)  China | Unknown | 1300 | Observational | ctDNA  CRC | NA | NA |
| (NCT02948985)  China | Unknown | 100 | Observational | ctDNA  mCRC | Adjuvant ChT | NA |
| OPTIPAL-II  (NCT03750175)  Denmark | Recruiting | 100 | Observational | ctDNA  CRC | NA | NA |
| ECLIPSE  (NCT04136002)  USA | Recruiting | 10000 | Observational | ctDNA  CRC | NA | NA |
| (NCT03189576)  Finland | Active, not recruiting | 37 | Observational | ctDNA  CRC | NA | NA |
| ADNCHIR  (NCT03809403)  France | Unknown | 40 | Observational | ctDNA  CRC | NA | NA |
| (NCT03868215)  China | Unknown | 100 | Observational | ctDNA  CRC | NA | NGS |
| (NCT03038217)  China | Unknown | 300 | Interventional | ctDNA  CRC | Adjuvant ChT | NGS |
| (NCT03803553)  USA | Recruiting | 500 | Interventional | ctDNA  mCRC | Adjuvant ChT | NA |
| (NCT03594448)  USA | Recruiting | 35 | Observational | ctDNA  mCRC | NA | NA |
| (NCT05127096)  USA | Recruiting | 1000 | Observational | ctDNA  CRC | NA | NA |
| (NCT04920032)  USA | Recruiting | 22 | Interventional | ctDNA  CRC | Drug: TAS-102, Irinotecan | NA |
| ALTAIR  (NCT04457297)  Japan | Recruiting | 240 | Interventional | ctDNA  CRC | Drug: trifluridine, tipiracil | NA |
| RASANC  (NCT02502656)  France | Unknown | 425 | Observational | ctDNA  CRC | NA | NA |
| IMPROVE-IT  (NCT03748680)  Denmark | Recruiting | 64 | Interventional | ctDNA  CRC | Adjuvant ChT | NGS |
| (NCT04385316)  China | Recruiting | 3 | Observational | ctDNA  CRC | NA | NGS |
| (NCT04735900)  Belgium | Recruiting | 60 | Interventional | ctDNA  mCRC | Adjuvant ChT | NA |
| (NCT03436563)  USA | Recruiting | 74 | Interventional | ctDNA  mCRC | Anti-PD-L1/TGF-beta RII Fusion Protein M7824 | NGS |
| SYNCOPE  (NCT04842006)  Finland | Not yet recruiting | 93 | Interventional | ctDNA  CRC | Adjuvant ChT | NA |
| (NCT04509635)  China | Not yet recruiting | 50 | Interventional | ctDNA  mCRC | Adjuvant ChT | NA |
| CircuLOR-1  (NCT02827565)  France | Completed | 30 | Interventional | ctDNA  mCRC | NA | NGS |
| PROSPECT-C  (NCT02994888)  UK | Completed | 47 | Interventional | ctDNA  mCRC | Drug: Cetuximab | NA |
| BESPOKE  (NCT04761783)  USA | Recruiting | 1539 | Observational | ctDNA  CRC and other tumors | NA | NA |
| Coca-Colon  (NCT01212510)  France | Completed | 200 | Interventional | ctDNA  mCRC | NA | NA |
| (NCT04739072)  USA | Recruiting | 1000 | Observational | ctDNA  CRC | NA | NA |
| (NCT04104633)  France | Recruiting | 20 | Interventional | ctDNA  CRC and breast cancer | NA | NA |
| (NCT03561350)  China | Unknown | 60 | Observational | ctDNA  CRC | NA | NA |
| (NCT05036109)  USA | Recruiting | 17 | Interventional | ctDNA  CRC | Drug: Aspirin, Vitamin D | NA |
| PARERE  (NCT04787341)  Italy | Recruiting | 214 | Interventional | ctDNA  CRC | Drug: Regorafenib, Panitumumab | NA |
| (NCT01983098)  France | Unknown | 100 | Observational | ctDNA  CRC | NA | NA |
| (NCT04555369)  China | Recruiting | 300 | Interventional | ctDNA  CRC | NA | NA |
| ALGECOLS  (NCT01198743)  France | Completed | 261 | Observational | ctDNA  CRC | NA | NGS |
| eDetect-mCRC  (NCT05068531)  Canada | Not yet recruiting | 100 | Observational | ctDNA  mCRC | NA | NA |
| PREDICT-IRFC  (NCT04767568)  France | Recruiting | 400 | Observational | ctDNA  CRC | NA | NA |
| COPE  (NCT04258137)  France | Recruiting | 332 | Interventional | ctDNA  CRC  NSCLC | NA | NA |
| (NCT04466267)  China | Completed | 40 | Observational | ctDNA  CRC | NA | NA |
| (NCT05040568)  USA | Not yet recruiting | 15 | Interventional | ctDNA  CRC | Drug: Cetuximab,  CB-NK-TGF-Î²R2-/NR3C1, Fludarabine phosphate, Cyclophosphamide | NA |
| COCA-MACS  (NCT02872779)  France | Unknown | 74 | Interventional | ctDNA  CRC | NA | NA |
| (NCT04230018)  China | Recruiting | 40 | Observational | ctDNA  mCRC | NA | NA |
| (NCT03223779)  USA | Recruiting | 56 | Interventional | ctDNA  CRC | Drug: TAS-102  Radiation: Photon SBRT | NA |
| (NCT05141721)  USA | Not yet recruiting | 665 | Interventional | ctDNA  CRC | Drug: GRT-C901,  GRT-R902,  Atezolizumab,  et al | NA |
| (NCT03829410)  USA | Recruiting | 44 | Interventional | ctDNA  mCRC | Adjuvant ChT | NA |
| CHRONOS  (NCT03227926)  Italy | Active, not recruiting | 129 | Interventional | ctDNA  CRC | Drug: Panitumumab | NGS |
| Toco-CoR  (NCT04245865)  Denmark | Recruiting | 74 | Interventional | ctDNA  mCRC | Adjuvant ChT | NA |
| (NCT03517332)  USA | Unknown | 10000 | Observational | ctDNA  CRC,  Other tumors | NA | NA |
| (NCT04247256)  USA | Recruiting | 35 | Interventional | ctDNA  mCRC | Adjuvant ChT  Drug: SCO-101 | NA |
| (NCT04587128)  USA | Recruiting | 110 | Interventional | ctDNA  mCRC | Drug: Panitumumab,  Cetuximab,  Irinotecan | NA |
| SIRT  (NCT04491929)  Denmark | Recruiting | 30 | Observational | ctDNA  mCRC | NA | NA |
| (NCT04929015)  USA | Active, not recruiting | 30 | Interventional | ctDNA  CRC | NA | NA |
| (NCT02838836)  USA | Recruiting | 120 | Observational | ctDNA  CRC,  Other tumors | NA | NA |
| AMPLIFY-201  (NCT04853017)  USA | Recruiting | 18 | Interventional | ctDNA  CRC | Drug: ELI-002 2P | NA |
| (NCT03526835)  USA | Unknown | 120 | Interventional | ctDNA  CRC,  Other tumors | Drug: MCLA-158 | NA |
| CR-SEQUENCE  (NCT03635021)  Spain | Recruiting | 370 | Interventional | ctDNA  CRC | Adjuvant ChT | NA |
| (NCT03542214)  Denmark | Completed | 6 | Interventional | ctDNA  Stage IV CRC | NA | NA |
| (NCT03487939)  China | Recruiting | 30 | Interventional | ctDNA  CRC | Adjuvant ChT | NA |
| (NCT04776837)  USA | Recruiting | 600 | Observational | ctDNA  CRC,  Other tumors | NA | NA |
| (NCT00730158)  USA | Completed | 33 | Interventional | ctDNA  CRC | Drug: KD018,  Irinotecan,  Placebo | NA |
| (NCT04169347)  USA | Recruiting | 35 | Interventional | ctDNA  mCRC | Drug: Oxaliplatin | NA |
| EXACT  (NCT03975491)  USA | Recruiting | 60 | Interventional | ctDNA  CRC | NA | NA |
| iRE-C  (NCT04108481)  USA | Recruiting | 18 | Interventional | ctDNA  mCRC | Drug: Durvalumab  Radiation: Yttrium-90 | NA |
| (NCT02848443)  Austria | Completed | 78 | Interventional | ctDNA  mCRC | Drug: Trifluridine/tipiracil hydrochloride (S 95005),  Oxaliplatin,  Bevacizumab,  Nivolumab | NA |
| (NCT02171286)  Canada | Completed | 432 | Observational | ctDNA  CRC,  Other tumors | NA | NA |
| LONGBOARD  (NCT04166604)  France | Recruiting | 250 | Interventional | ctDNA  mCRC | Drug: Trifluridine/Tipiracil | NA |
| (NCT03841799)  France | Recruiting | 80 | Observational | ctDNA  CRC | NA | NA |
| KISIMA-01  (NCT04046445)  USA | Recruiting | 96 | Interventional | ctDNA  mCRC | Drug: ATP128,  BI 754091,  VSV-GP128 | NA |
| IMPROVE  (NCT04425239)  Italy | Active, not recruiting | 151 | Interventional | ctDNA  mCRC | Drug: Panitumumab | NA |
| PembroMab  (NCT02318901)  USA | Terminated  Has Results | 16 | Interventional | ctDNA  CRC,  Other tumors | Drug: Pembrolizumab,  Trastuzumab,  ado-trastuzumab emtansine,  Cetuximab | NA |
| EXOSCOL01  (NCT04394572)  France | Recruiting | 80 | Observational | Exosomes  CRC | NA | NA |
| ExoColon  (NCT04523389)  France | Recruiting | 172 | Observational | Exosomes  CRC | NA | NA |
| NCT03927898  China | Recruiting | 40 | Interventional | Exosomes  mCRC | Adjuvant ChT  Radiotherapy | NA |
| NCT03260179  China | Unknown | 60 | Interventional | Exosomes  mCRC | Drug: AL3810 | NA |
| NCT02439008  France | Terminated  No Results | 28 | Interventional | Exosomes  mCRC  other tumors | Radiotherapy | NA |
| NCT01294072  USA | Recruiting | 35 | Interventional | Exosomes  Colon cancer | NA | NA |
| NCT03874559  USA | Recruiting | 30 | Observational | Exosomes  Rectal cancer | NA | NA |

CTC: circulating tumor cell; ctDNA: circulating tumor DNA; ChT: Chemotherapy; FCM: Flow cytometry; NGS: Next generation sequencing; Safe-SeqS: Safe-Sequencing System; ddPCR: droplet digital PCR;
